# Long non-coding RNA TFAP2A-AS1 plays an important role in oral squamous cell carcinoma: research includes bioinformatics analysis and experiments

**DOI:** 10.1186/s12903-022-02203-4

**Published:** 2022-05-06

**Authors:** Guo Jie, ShiXiong Peng, ZiFeng Cui, Chen He, XuPo Feng, Kaicheng yang

**Affiliations:** 1 Department of Stomatology, The Fourth Hospital of Hebei Medical University, No. 12 Jiankang Road, Shijiazhuang, 050000 Hebei Province China; 2 Department of Stomatology, Zhao County People’s Hospital, No. 1 Yongtong Road, Shijiazhuang, 050000 Hebei Province China

**Keywords:** Oral squamous cell carcinoma, TFAP2A-AS1, Bioinformatics, WGCNA

## Abstract

**Background:**

Oral squamous cell carcinoma (OSCC) is the most common neck and head malignancies, and the prognosis is not good. Studies shown that the long non-coding RNA (lncRNA) TFAP2A-AS1 is involved in the progression of multiple cancers. However, the role of lncRNA TFAP2A-AS1 in OSCC remains unclear. We aimed to explore the functions and expression in OSCC.

**Methods:**

The lncRNA profiles for OSCC patients were acquired from the TCGA. Based on these data, the data mining of TFAP2A-AS1 in patients with OSCC were performed. The functions of TFAP2A-AS1 were determined by bioinformatics analysis. The expression and roles in cell growth were tested by RT-qPCR and MTS assay. Cell invasion and migration were tested by wound healing and transwell assays.

**Results:**

The consequences displayed that TFAP2A-AS1 was upregulated in the TCGA datasets. The expression of TFAP2A-AS1 was higher in OSCC samples. Bioinformatics analysis shown that TFAP2A-AS1 might be associated with the P53 signaling pathway. Cell culture experiments indicated that deficiency of TFAP2A-AS1 inhibited cell growth, invasion, and migration, and overexpression of it could opposite results in SCC-25 cells.

**Conclusion:**

The results suggested that TFAP2A-AS1 was overexpressed in OSCC cells, which could facilitate OSCC cell proliferation, migration, and invasion.

**Supplementary Information:**

The online version contains supplementary material available at 10.1186/s12903-022-02203-4.

## Background

At present, Oral squamous cell carcinoma (OSCC) is creating a shocking situation worldwide, which is a major public health problem [1]. It has been reported that approximately 350,000 new cases of OSCC are diagnosed every year, which are responsible for approximately 170,000 deaths worldwide per year [2]. The morbidity and mortality of OSCC constitute a major health problem in many areas of the world. Moreover, studies have shown that OSCC is often diagnosed in the advanced stage of the disease (metastasis or advanced regional disease), which hinders the favorable outcomes of patients with OSCC [3, 4]. Patients with OSCC are managed with surgery, radiation, and chemotherapy. In recent years, despite great improvements in cancer surgical techniques and adjuvant therapy, the 5-year overall survival rate of patients with OSCC is still dismal [5, 6]. Therefore, it is required to find new molecular markers for diagnosis and treatment of OSCC.

Long non-coding RNAs (lncRNAs) are RNAs longer than 200 nt in length, which can regulate physiological and pathological processes by promoting target mRNA degradation or inhibiting target mRNA translation. It has been reported that lncRNAs are closely associated with tumorigenesis and progression of multiple cancers [7, 8]. Moreover, studies have shown that many lncRNAs are differentially expressed in OSCC, and some of them can modulate OSCC development [9, 10]. TFAP2A-AS1 (TFAP2A antisense RNA 1) is a novel lncRNA with unclear functions located on chromosome 6q24.3. Zhao et al. indicated that TFAP2A-AS1 was significantly associated with overall survival in bladder cancer patients, and TFAP2A-AS1 was a protective factor against bladder cancer [11]. Zhou et al. indicated that TFAP2A-AS1 could inhibit breast cancer development by functioning as an miR-933 sponge and degrading SMAD2 mRNA [12]. However, Jiang et al. reported that high expression of TFAP2A-AS1 could predict poor prognosis in patients with clear cell renal cell carcinoma [13]. However, the role of lncRNA TFAP2A-AS1 in OSCC remains unclear.

In this study, the expression and functions of TFAP2A-AS1 in OSCC cells were investigated by data mining and experiments.

## Materials and methods

### OSCC data source and reprocessing

The lncRNA profiles was got from the UCSC database (http://xena.ucsc.edu/) Ensembl IDs were transferred into gene symbols based on the GENCODE project gene annotation file (version 22, GRCh38). Then, these data were normalized and summarized by R packages “affy” and “limma.” The differential expression analysis was performed by R packages “limma” and the original P value is adjusted by the Benjamini–Hochberg method.

### WGCNA

The modules of highly correlated genes and the correlation was calculated by WGCNA. R package “WGCNA” (http://www.genetics.ucla.edu/labs/horvath/CoexpressionNetwork/Rpackages/WGCNA) was employed for WGCNA. First, the availability of genes in the RNA-seq data was calculated, and an adjacency matrix was constructed. Then, the correlation strength between genes was identified using the adjacency matrix. Then, the adjacency matrix was used to conduct the topological overlap matrix (TOM). The gene modules were identified by hierarchical clustering, and similar modules were merged (threshold = 0.25). Furthermore, the significant modules were derermined.

### Gene set enrichment analysis

Gene set enrichment analysis (GSEA) is a computational method that calculates whether a previously defined gene set shows a statistically significant difference. GSEA analysis was used to explore the functions of TFAP2A-AS1. According to the median expression value of TFAP2A-AS1, OSCC samples were divided into high expression group and low expression group. GSEA software (http://www.gsea-msigdb.org/gsea/index.jsp) was used to perform GSEA. |NES|> 1 and FDR < 0.05 were considered statistically significant.

### Tissue samples and cell lines

The OSCC tissue and adjacent normal tissues were got from our hospital (The Fourth Hospital of Hebei Medical University, Shijiazhuang, China). The inclusion criteria for this study are as follows: (1) Oral squamous cell carcinoma confirmed by pathology before surgery, and can be treated by surgery (2) Patients who have not received radiotherapy or chemotherapy before surgery (3) Patients who have signed informed consent. The exclusion criteria for this study are as follows: (1) Patients with oral squamous cell carcinoma who cannot be treated by surgery (2) Patients who received radiotherapy or chemotherapy before surgery (3) Patients who did not sign the informed consent form (4) Patients with incomplete clinical data. Informed consent was collected from all patients. This study was approved by the local ethics committee and carried out in compliance with the Declaration of Helsinki. The OSCC cell lines and normal control were got from the pathology research room in our hospital (The Fourth Hospital of Hebei Medical University, Shijiazhuang, China).

### RT-qPCR assay

RT-qPCR was performed to measure the expression of TFAP2A-AS1. Firstly, the total RNA was extracted by TRIzol reagent (West Point Chemical Technology Co., Ltd., Tianjin, China). For TFAP2A-AS1 and GAPDH expression analysis, RNA was used to synthesize complementary DNA (cDNA) using a Reverse Transcription Kit (Roche, Basel, Switzerland). The experiments were performed three times, and data were calculated using the 2 − ΔΔCt method. The primers were shown in Additional file 1: Table S1.

### Construction of vectors and cell transfection

In this study, the TFAP2A-AS1 specific shRNA vectors and their controls were acquired from Gemma Gene Corporation (Shanghai, China). Vectors were transfected into OSCC cell lines using Lipofectamine® RNAiMAX Reagent (Thermo Fisher Scientific, Inc., Waltham, MA, USA) according to the manufacturer's protocol. The shRNA sequences for TFAP2A-AS1 were sh1-1: 5′- GCCGCTCCGACACAGGTATAA -3′, sh1-2: 5′-GGCCCAGCTTCTAGCACTTCT-3′, sh1-3: 5′- GGGTTTGCAATCCCTTAATTG-3′, sh1-4: 5′-GGGCCAACTGACAGTTCAAAT-3′, sh1-5: 5′-GCAAGTCTAGATACCGTCTCG-3′, sh-NC: 5′-AATTCTCCGAACGTGTCACGT-3′. The TFAP2A-AS1 control and overexpression (OE) plasmid were obtained from Huifeng Biological Technology Co., Ltd (Changsha, China). Transfection Reagent FuGENE® (West Point Chemical Technology Co., Ltd.) was used for plasmid transfection.

### MTS assay

Cell viability was assessed using the CellTiter 96® AQueous One Solution Cell Proliferation Assay Kit (Promega). For TFAP2A-AS1downregulation, the cells were divided into sh-TFAP2A-AS1 and sh-normal control (NC) groups. For TFAP2A-AS1 overexpression, the cells were divided into ov-TFAP2A-AS1group and ov-NC groups. Cultivate OSCC cells for a period of time. Then add 20 μl MTS reagent and incubate at 37 °C for 2 h. The absorbance was measured at 492 nm.

### Colony formation assay

For TFAP2A-AS1 downregulation, the cells were divided into sh-TFAP2A-AS1 and sh-normal control (NC) groups. For TFAP2A-AS1 overexpression, the cells were divided into ov-TFAP2A-AS1group and ov-NC groups. OSCC cells (3 × 10^3^) were cultured for 1 week. 4% paraformaldehyde was used to fix cells. Furthermore, the colonies were stained. OSCC cells were counted to assess cell proliferation.

### Wound healing assay

For TFAP2A-AS1 downregulation, the cells were divided into sh-TFAP2A-AS1 and sh-normal control (NC) groups. For TFAP2A-AS1 overexpression, the cells were divided into ov-TFAP2A-AS1group and ov-NC groups. OSCC cells (5 × 10^5^) were seeded in 6-well plates. Then, the pipette tip was used to created wound, and the photos were captured after this. Each group repeated the process three times.

### Transwell assay

For TFAP2A-AS1downregulation, the cells were divided into sh-TFAP2A-AS1 and sh-normal control (NC) groups. For TFAP2A-AS1 overexpression, the cells were divided into ov-TFAP2A-AS1 group and ov-NC groups. Transwell assays were performed using 24-well transwell chambers (Corning Incorporated, Corning, NY, USA) with Matrigel-coated membranes. Serum-free medium was used to inoculate OSCC cells in the upper chamber for 24 h. Then, 4% paraformaldehyde was used for fixing cells and 0.1% crystal violet was used for staining cells.

### Xenograft mice model of OSCC

In this study, the xenograft mice model was carried out in accordance with the requirements of the Ethics Committee of the Fourth Hospital of Hebei Medical University. The BALB/c nude mice were purchased from Cosmo Biotechnology Co., Ltd. (Tianjin, China). The sh-TFAP2A-AS1 OSCC cells, ov-TFAP2A-AS1 and NC OSCC cells were suspended in 100 μL medium (1 × 10^7^). Then the OSCC cells were injected into the right flanks of mice. Tumor growth was measured at 3, 5, 7, 9, 11,13 and 15 days after injection. 15 days after injection, all mice were euthanized with sedatives and overdose of anesthesia. And tumor weights were measured.

### Statistical analysis

In this study, all data were analyzed using R software (version 4.0.5) and SPSS 24.0 (IBM, Armonk, NY, USA). For normally distributed data, comparisons between groups were made using the t-test. For data that did not follow a normal distribution, nonparametric tests were used for comparisons between groups. The OS of patients was explored by Kaplan–Meier method. The performance of TFAP2A-AS1 was evaluated using ROC curve analysis. For correlations between TFAP2A-AS1 and OSCC: (1) For all theoretical numbers T ≥ 5 and total sample size n ≥ 40, use Pearson's chi-square test. (2) If theoretical numbers T < 5 but T ≥ 1, and n ≥ 40, use a continuity-corrected chi-square test (3) If there is a theoretical number T < 1 or n < 40, use Fisher's test. The multivariate cox analysis was performed to explore the effect of TFAP2A-AS1on survival of the patient in UCSC database. Statistical significance was set at *P* < 0.05. All experiments were performed in triplicate.

## Results

### Data mining of TFAP2A-AS1

**Fig. 1:**
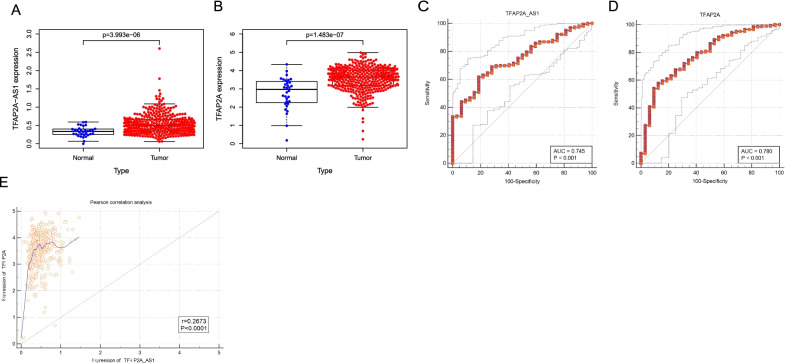
Differential expression analysis of the genes in the TCGA database. **A** Box diagram of TFAP2A-AS1 showing differential expression between OSCC and normal groups. **B** Box diagram of TFAP2A showing differential expression between OSCC and normal groups. **C** ROC analysis of TFAP2A-AS1. **D** ROC analysis of TFAP2A. **E** Pearson correlation analysis between TFAP2A-AS1 and TFAP2A

The RNA-seq data and corresponding clinicopathological data of OSCC patients were downloaded and reanalyzed. TFAP2A-AS1 was upregulated in the TCGA-OSCC datasets (Fig. 1A, *P* = 3.993e−06). TFAP2A is located on the other chain of TFAP2A-AS1. Thus, the expression of TFAP2A was analyzed. TFAP2A was also overexpressed in the TCGA-OSCC datasets (Fig. 1B, *P* = 1.483e-07). For TFAP2A-AS1, the area under curve was 0.745 (Fig. 1C, *P* < 0.001), and 0.780 for TFAP2A. (Fig. 1D, *P* < 0.001), suggesting that TFAP2A-AS1 and TFAP2A might be diagnostic biomarkers for OSCC. As shown in Fig. 1E, TFAP2A-AS1 expression was positively correlated with TFAP2A expression (r = 0.1454, *P* = 0.0072). However, multivariate cox analysis suggested that TFAP2A-AS1 was not associated with overall survival of patients (Table 1, *P* = 0.680).

**Table 1:** Correlative effect on survival of the patient based on multivariate cox analysis

Characteristics	SSI		
	OR	95% CI	*P*
TFAP2A-AS1	1.143	0.606–2.159	0.680
Age	1.028	1.013–1.043	0.000
Gender	1.029	0.723–1.463	0.874
T stage	1.270	0.957–1.686	0.098
N stage	1.224	0.954–1.569	0.112
M stage	6.572	1.440–30.001	0.015
TNM stage	0.841	0.583–1.214	0.355

OR, odds ratio; 95% CI, 95% confidence interval

### WGCNA

**Fig. 2:**
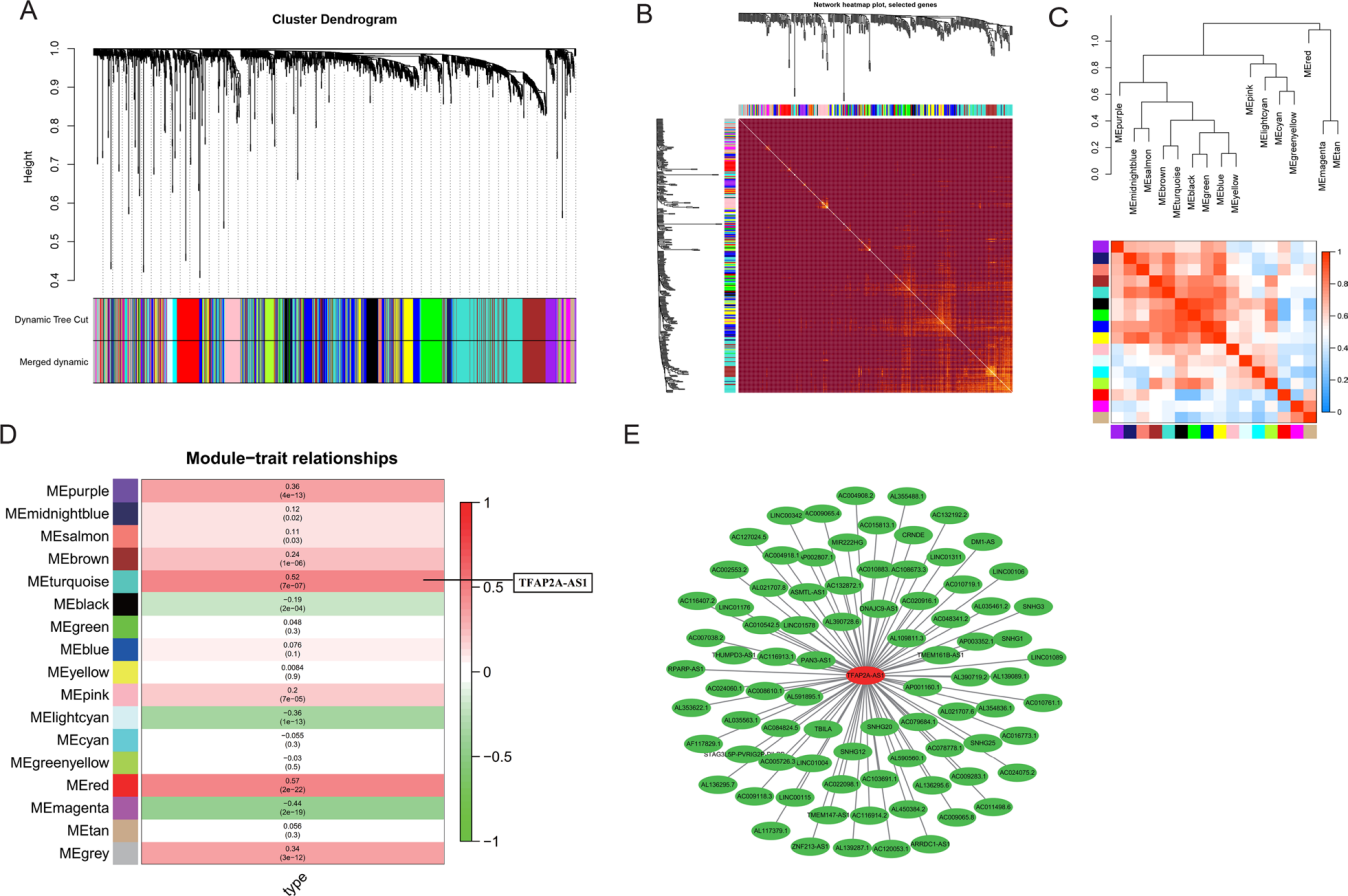
WGCNA analysis of the genes in the TCGA database. **A**, **B** Repeated hierarchical clustering tree of all genes and TFAP2A-AS1is in the turquoise module. **C** The dendrogram and heatmap of all genes. **D** Interactions between these modules. **E** The associations between clinic traits and the modules and the correlation between turquoise module and OSCC is 0.52. **F** Interrelationships between TFAP2A-AS1and the genes in the turquoise module

The OSCC-related modules were determined by WGCNA. As shown in Fig. 2A, a hierarchical tree of RNA-seq data was constructed and dendrogram of it was shown in Fig. 2B. The correlation heatmap of the modules is shown in Fig. 2C. The dendrogram and correlation heatmap suggested that there was no significant difference in the interaction. As shown in Fig. 2D, the TFAP2A-AS1-related module are highly correlated with OSCC. The lncRNAs in the TFAP2A-AS1-related module were obtained (Fig. 2E), which showed similar gene expression patterns to TFAP2A-AS1.

### GSEA analysis

**Fig. 3:**
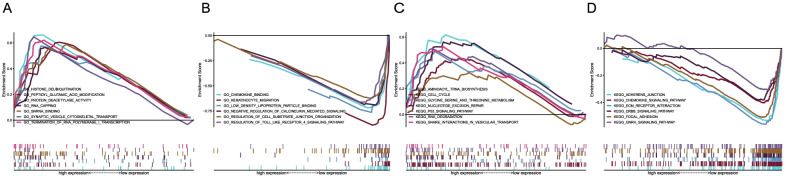
Gene functional enrichment analysis of TFAP2A-AS1. **A**, **B** GO analyses by GSEA. **C**, **D** KEGG analyses of by GSEA

GO method indicated that histone deubiquitination, peptidyl glutamic acid modification, and protein deacetylase activity were upregulated (Fig. 3A), whereas chemokine binding, keratinocyte migration, and low-density lipoprotein particle binding were downregulated (Fig. 3B). For KEGG (https://www.kegg.jp/) analysis, cell cycle, P53 signaling pathway, RNA degradation, and so on were upregulated in OSCC (Fig. 3C), whereas adherens junction, chemokine signaling pathway, ERBB signaling pathway, and so on were downregulated (Fig. 3D).

### The expression of TFAP2A-AS1

**Fig. 4:**
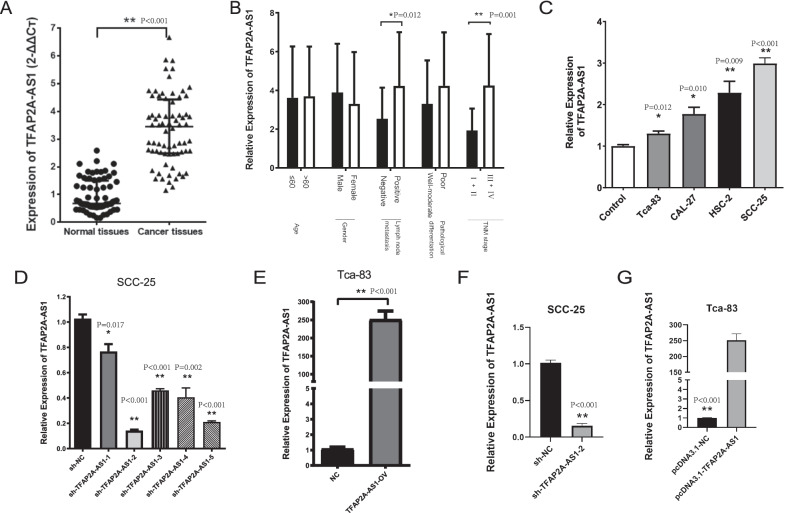
The expression of TFAP2A-AS1. **A** Relative expression levels of TFAP2A-AS1 in the Clinical OSCC samples which included 64 OSCC tissues and 64 paired tissues from the adjacent normal tissues. **B** The relationship between the expression of TFAP2A-AS1 and clinicopathologic features of OSCC tissues **C** The expression of TFAP2A-AS1 in OSCC cell lines. **D** Validation of TFAP2A-AS1 knock down in OSCC cell lines. **E** Validation of TFAP2A-AS1 over-expression in OSCC cell lines. **F** Validation of TFAP2A-AS1 knock down in OSCC cell lines 15 days after transfection. **G** Validation of TFAP2A-AS1 over-expression in OSCC cell lines 15 days after transfection. *P < 0.05 and **P < 0.01

In our study, the expression level of TFAP2A-AS1 was higher in cancer tissues than in adjacent normal tissues (Fig. 4A, *P* < 0.01, Mann Whitney test). Correlations between TFAP2A-AS1 and clinicopathological characteristics (i.e., age, sex, lymphatic metastasis, degree of organizational differentiation, and TNM stage) was calculated (Table 2). TFAP2A-AS1 related to lymphatic metastasis and TNM stage. Moreover, as shown in Fig. 4B, TFAP2A-AS1 expression was higher in lymphatic metastasis than in no metastasis (*P* = 0.012, Table 2, t-test) and higher in TNM stages III and IV than in stages I and II (*P* = 0.001, Table 2, t-test). As shown in Fig. 4C, the expression level of TFAP2A-AS1 was higher in OSCC cell lines than in NHOK cells. The expression level of TFAP2A-AS1 was the highest in SCC-25 cells (*P* < 0.001, t-test) and the lowest in Tca-83 cells (*P* = 0.012, t-test). Thus, SCC-25 cells were used for knockdown experiments, and Tca-83 cells were used for overexpression experiments. As shown in Fig. 4D, transfection with sh-TFAP2A-AS1 significantly downregulated TFAP2A-AS1 in SCC-25 cells. Among them, the sh2 (*P* < 0.001, t-test) knockdown site had the best transfection efficiency, so the sh2 knockdown site was selected for subsequent experiments. Moreover, transfection with TFAP2A-AS1 vectors significantly upregulated TFAP2A-AS1 in Tca-83 cells (Fig. 4E, *P* < 0.001, t-test). Moreover, the persistent effects of sh-TFAP2A-AS1 and TFAP2A-AS1 vectors on cells was detected. As shown in Fig. 4F, G, the effect of both sh-TFAP2A-AS1 (*P* < 0.001, t-test) and TFAP2A-AS1 vectors (*P* < 0.001, t-test) for TFAP2A-AS1 is persist.

**Table 2:** Clinicopathological variables and the expression of TFAP2A-AS1

Parameters	N	TFAP2A-AS1		
		$\overline{x}$ ± s	*t*	*P*-value
*Age/year*				
≤ 60	24	3.626 ± 2.637	− 0.114	0.910
> 60	40	3.702 ± 2.557		
*Gender*				
Male	40	3.894 ± 2.510	0.887	0.379
Female	24	3.305 ± 2.670		
*Lymphatic metastasis*				
N0	21	2.531 ± 1.614	− 2.599	0.012
N1–N2	43	4.231 ± 2.769		
*Organizational differentiation*				
Moderate and high	36	3.312 ± 2.227	− 1.500	0.139
Poorly	28	4.245 ± 2.748		
*TNM stage*				
I	5	1.927 ± 1.138	6.714	0.001
II	11			
III	38	4.255 ± 2.652		
IV	10			
Total	64	3.673 ± 2.566	6.492	0.000

### TFAP2A-AS1 promoted OSCC cell development

**Fig. 5:**
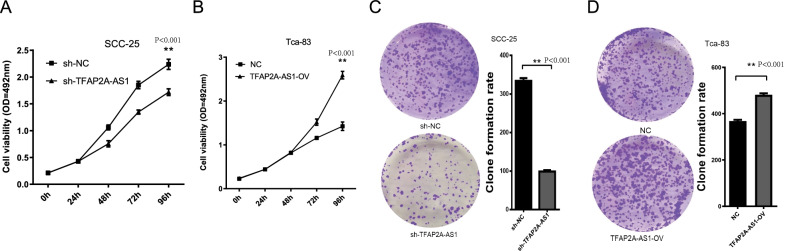
MTS and CCK-8 analysis are used to study the effect of knocking down or overexpression of TFAP2A-AS1 on cell proliferation in the cell line. **A** The results of MTS assay in TFAP2A-AS1 knocked down cell line. **B** The results of MTS assay in TFAP2A-AS1 over-expression cell line. **C** The results of clone formation experiment of TFAP2A-AS1 knocked down cell line. **D** The results of clone formation experiment of TFAP2A-AS1 over-expression cell line

**Fig. 6:**
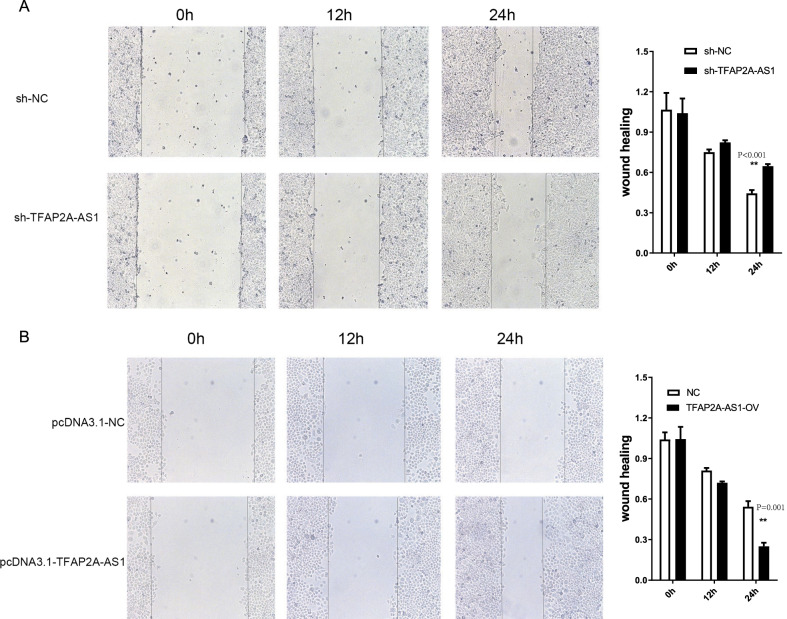
Wound healing assay of TFAP2A-AS1. **A** Wound healing assay of TFAP2A-AS1 knock down in OSCC cell lines. **B** Wound healing assay of TFAP2A-AS1 over-expression in OSCC cell lines. The Y-axis represents cell migration distance

**Fig. 7:**
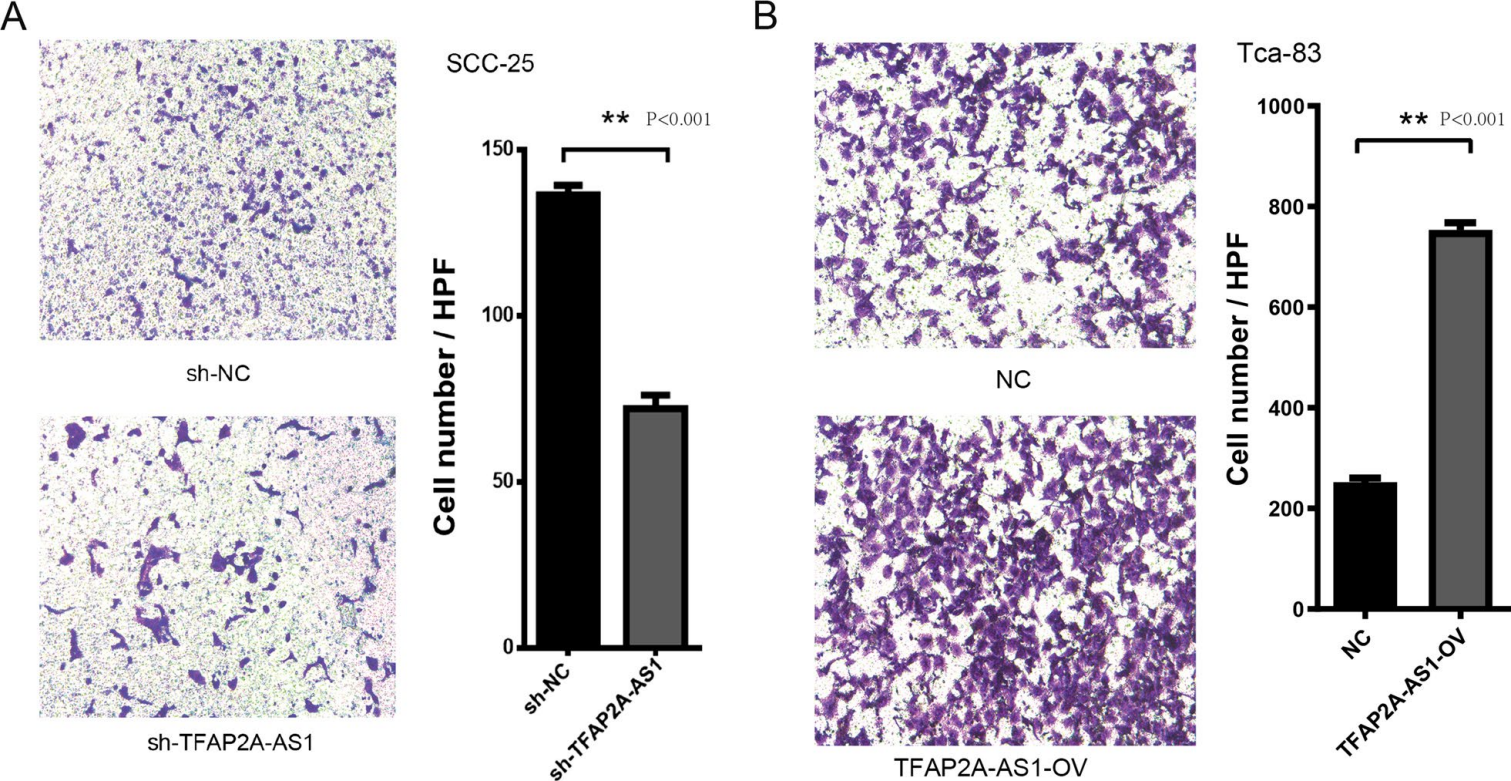
Transwell assay of TFAP2A-AS1. **A** Transwell assay of TFAP2A-AS1 knock down in OSCC cell lines. **B** Transwell assay of TFAP2A-AS1 over-expression in OSCC cell lines

**Fig. 8:**
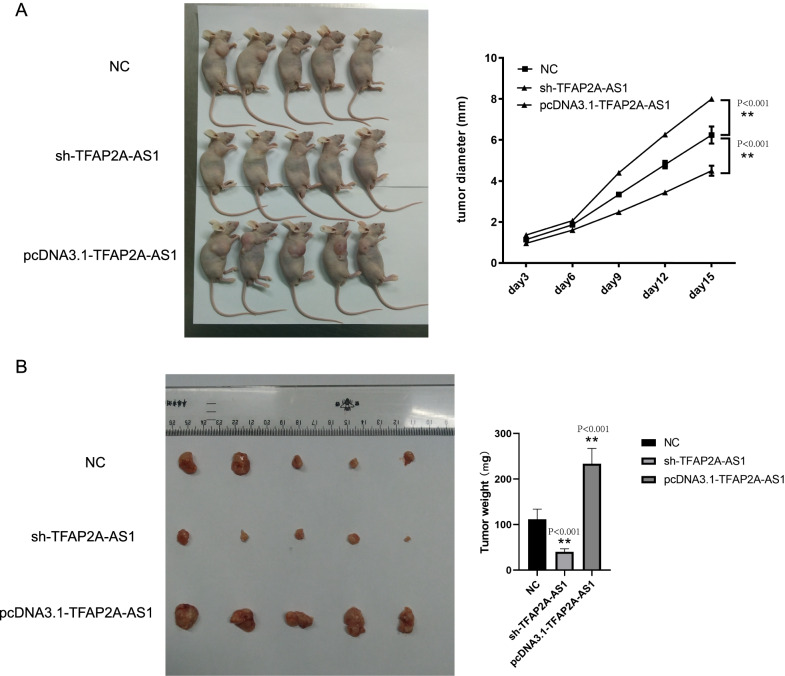
Effect of TFAP2A-AS1 knockdownon or overexpression on the growth (**A**) and weight (**B**) of xenografts derived from OSCC cells. **P < 0.001

The MTS assay was performed to assess the cell viability. As shown in Fig. 5A, the proliferation of SCC-25 cells was noticeably inhibited after transfection with sh-TFAP2A-AS1 (P < 0.001, t-test), whereas transfection with TFAP2A-AS1 vectors noticeably promoted the growth of Tca-83 cells at 72 and 96 h (Fig. 5B, *P* < 0.001, t-test). Moreover, a colony formation assay was used to analyze cell growth. Transfection with sh-TFAP2A-AS1 significantly suppressed colony formation, whereas TFAP2A-AS1 overexpression increased colony formation (Fig. 5C, D, *P* < 0.001, t-test). Wound healing and transwell assays were used to identify the effects of TFAP2A-AS1 on cell migration and invasion. TFAP2A-AS1 knockdown reduced the SCC-25 migratory capacity (Fig. 6A, *P* < 0.001, t-test), whereas TFAP2A-AS1 overexpression significantly enhanced the migratory capacity of Tca-83 cells (Fig. 6B, *P* < 0.001, t-test). In the Transwell assay, TFAP2A-AS1 knockdown significantly decrease the number of SCC-25 cells stained with crystal violet (Fig. 7, *P* < 0.001, t-test), while TFAP2A-AS1 overexpression significantly enhanced the invasive capacity of Tca-83 cells (Fig. 7B, *P* < 0.001, t-test). The xenograft mice model of OSCC was performed to further validate our conclusion. As shown in Fig. 8, the TFAP2A-AS1 knockdown inhibited the tumor growth (Fig. 8A, *P* < 0.001, t-test), whereas TFAP2A-AS1 overexpression significantly enhanced the tumor growth (Fig. 8B, *P* < 0.001, t-test). All these data indicate that TFAP2A-AS1 could promoted OSCC cell development.

## Discussion

Oral cancer is considered a serious health problem, leading to high morbidity and mortality rates. OSCC was a frequent subtype of oral cancer and is the major cause of morbidity and mortality [5, 14]. It has been reported that visual examination is the main method for diagnosing oral cancer, which may lead to delayed diagnosis [15, 16]. The five-year survival rate of OSCC is still low, and delayed diagnosis is considered one of the main reasons. Currently, the treatment methods for OSCC patients include surgery, chemoradiotherapy, EGFR inhibitors, COX-2 inhibitors, and photodynamic therapy [17]. Despite some progress has been made in the treatment of OSCC in recent years, the survival rate remains low [18]. Therefore, it is required to find new molecular markers for diagnosis and treatment of OSCC.

Misregulation and mutations of lncRNAs play important roles in cancer. Dinescu et al. indicated that alterations and mutations in lncRNA expression could promote tumorigenesis and metastasis [19]. Bhan et al. indicated that lncRNAs could inhibit or promote the development of cancer cells [7]. Studies have shown that dysregulation of many lncRNAs was involved in cellular homeostasis, including survival, migration, and genomic stability of OSCC [9, 10]. Thus, lncRNAs have a strong potential as new biomarkers and cancer treatment targets. It has been reported that TFAP2A-AS1 play a critical role in the tumorigenesis and development of breast and bladder cancers [11, 12]. Therefore, we believe that TFAP2A-AS1 may play an important role in the occurrence and development of OSCC.

TFAP2A-AS1 was overexpressed in TCGA-OSCC datasets. Moreover, TFAP2A-AS1 was upregulated in OSCC cell lines and tissue samples. Furthermore, its expression related to lymphatic metastasis and TNM stage. The deficiency of TFAP2A-AS1 could inhibit OSCC cell progression and overexpression of it promoted cell progression. These results suggest that TFAP2A-AS1 may be used as an oncogene that influences the malignant biological behavior of OSCC.

In our study, lncRNAs in the TFAP2A-AS1-related module were obtained. ASMTl-AS1 (ASMTL antisense RNA 1) serves as an antisense to the acetylserotonin O-methyltransferase-like gene. It has been reported that it is significantly downregulated in papillary thyroid carcinoma, which is associated with tumor size, clinical stage, and outcome. Moreover, knockdown of ASMTL-AS1 promoted PTC cell proliferation and glycolysis [20]. Sun et al. indicated that ASMTL-AS1 was significantly downregulated in triple-negative breast cancer, which is associated with aggressive clinical features and unfavorable prognosis [21]. Therefore, we believe that ASMTL-AS1 is related to the development of OSCC; however, further experiments are needed to prove this idea. Small nucleolar RNA host gene 12 (SNHG12) produces a long RNA that is overexpressed in cancer cells. Liu et al. reported that SNHG12 was upregulated in renal cell carcinoma (RCC) tissues and could predict poor prognosis. Moreover, SNHG12 could promote development and sunitinib resistance in RCC cells [22]. Tamang et al. indicated that SNHG12 is abnormally expressed in many cancers. Moreover, SNHG12 is involved in unfolded protein responses, which many tumor cells use to evade immune-mediated attacks and enhance the polarization of effector immune cells [23]. Thus, SNHG12 may serve as both a biomarker and a therapeutic target for patients with OSCC. Colorectal neoplasia differentially expressed (CRNDE) is transcribed into multiple transcript variants and is increased in proliferating tissues, including colorectal adenomas and adenocarcinomas. It has been reported that CRNDE knockdown in cell lines could inhibit the Wnt/β-catenin signaling pathway, which leads to the inhibition of cell proliferation and reduction of chemoresistance [24]. Meng et al. indicated that CRNDE was overexpression in cervical cancer, and CRNDE can promote cell growth and metastasis of cervical cells [25]. CRNDE was found in TFAP2A-AS1-related module and we surmised that CRNDE may also play an important in OSCC.

Cell proliferation is a key feature of OSCC development. In this study, the MTS assay and colony formation assay indicated that TFAP2A-AS1 promoted cell proliferation in OSCC cells. The P53 signaling pathway was enriched in this study. The P53 signaling pathway plays an important role in the development of cancer cells [26–28]. Wei et al. indicated that lncRNA MEG3 decreased the gastric cancer cell growth via the P53 signaling pathway [29]. Moreover, Xing et al. indicated that TNFAIP8 could promote the proliferation of non-small cell lung cancer cells through the p53 signaling pathway [30]. Therefore, we believed that TFAP2A-AS1 promotes the proliferation via the P53 signaling pathway. Our study indicated that TFAP2A-AS1 promoted cell invasion in OSCC cells. Liu et al. indicated that RECK could suppress the migration and invasion of cervical cancer cells via the p53 signaling pathway [31]. Moreover, Zhong et al. reported that Cullin-4B promotes colorectal cancer cell invasion through inactivation of the p53 signaling pathway [32]. Furthermore, the ERBB signaling pathway was also enriched. Dermatan sulfate epimerase 1 promotes aggressive glioma cell phenotypes by enhancing the ERBB signaling pathway [33]. Therefore, we surmised that TFAP2A-AS1 promotes OSCC cell invasion through the P53 and ERBB signaling pathways. However, these results need to be examined in future studies.

This study has some limitations, such as insufficient sample size, only one cell line was used in part of the experiment, and the pathway obtained in GSEA has not been experimentally verified. In the follow-up research process, we will conduct more research to solve these problems.

## Conclusion: TFAP2A-AS1 function as an oncogene in OSCC

In short, we demonstrated that TFAP2A-AS1 has a tumor-promoting effect in OSCC cells, which could be molecular markers for diagnosis and treatment of OSCC.

## Supplementary Information

**Additional file 1**: **Table S1.** The Primers and sequence in RT-qPCR assay.

## Data Availability

The datasets analysed during the current study are available in the UCSC repository, [http://xena.ucsc.edu/ and cohort: GDC TCGA Head and Neck Cancer (HNSC)].
