# Supplementary material for: Long non-coding RNA TFAP2A-AS1 plays an important role in oral squamous cell carcinoma: research includes bioinformatics analysis and experiments
Source: BMC Oral Health. 2022 May 6;22:160. doi: 10.1186/s12903-022-02203-4 (PMC9074241; doi:10.1186/s12903-022-02203-4)
Supplement: Supplementary file 1 — Additional file 1: Table S1. The Primers and sequence in RT-qPCR assay. [file 12903_2022_2203_MOESM1_ESM.docx]

Table.S1

| Primers | sequence |
| --- | --- |
| TFAP2A-AS1 | forward, 5′- CCTGTGACCGCACGGATGAT -3′, reverse, 5’- CGAGACCGAGAGGGGCATAT -3’ |
| GAPDH | forward, 5’-TGTGTCCGTCGTGGATCTGA-3’, reverse, 5’-CCTGCTTCACCACCTTCTTGA-3’ |
|  |  |

Table.S1 The Primers and sequence in RT-qPCR assay.
